# The ADAMs family of proteases: new biomarkers and therapeutic targets for cancer?

**DOI:** 10.1186/1559-0275-8-9

**Published:** 2011-06-09

**Authors:** Michael J Duffy, Maeve Mullooly, Norma O'Donovan, Sumainizah Sukor, John Crown, Aisling Pierce, Patricia M McGowan

**Affiliations:** 1 Department of Pathology and Laboratory Medicine, St. Vincent's University Hospital, Dublin 4, Ireland; 2 UCD School of Medicine and Medical Science, Conway Institute of Biomolecular and Biomedical Research, University College Dublin, Dublin 4, Ireland; 3 National Institute for Cellular Biotechnology, Dublin City University, Dublin 9, Ireland; 4 Department of Medical Oncology, St Vincent's University Hospital, Dublin 4, Ireland

## Abstract

The ADAMs are transmembrane proteins implicated in proteolysis and cell adhesion. Forty gene members of the family have been identified, of which 21 are believed to be functional in humans. As proteases, their main substrates are the ectodomains of other transmembrane proteins. These substrates include precursor forms of growth factors, cytokines, growth factor receptors, cytokine receptors and several different types of adhesion molecules. Although altered expression of specific ADAMs has been implicated in different diseases, their best-documented role is in cancer formation and progression. ADAMs shown to play a role in cancer include ADAM9, ADAM10, ADAM12, ADAM15 and ADAM17. Two of the ADAMs, i.e., ADAM10 and 17 appear to promote cancer progression by releasing HER/EGFR ligands. The released ligands activate HER/EGFR signalling that culminates in increased cell proliferation, migration and survival. Consistent with a causative role in cancer, several ADAMs are emerging as potential cancer biomarkers for aiding cancer diagnosis and predicting patient outcome. Furthermore, a number of selective ADAM inhibitors, especially against ADAM10 and ADAM17, have been shown to have anti-cancer effects. At least one of these inhibitors is now undergoing clinical trials in patients with breast cancer.

## Review

The ADAMs are a family of multidomain proteins shown to be involved in both proteolysis and cell adhesion [for review, see refs [1-3]]. Although primarily located on the cell membrane, soluble forms have been described for some ADAMs. The best established role for ADAMs is the activation of the proforms of certain growth factors and cytokines as well as the shedding of the extracellular domains of growth factor receptors and adhesion proteins. ADAMs thus play a role in remodelling or processing of cell membrane proteins. Several of the substrates processed by ADAMs, especially by ADAM10 and ADAM17, have been implicated in the pathogenesis or progression of cancer [for reviews, see refs [4,5]], though some proteolytically inactive ADAMs may also play important roles in carcinogenesis (summarised in Table 1). The aim of this article is to review the role of ADAMs in malignancy, focusing especially on their potential use as cancer biomarkers and therapeutic targets. Firstly however, we briefly review the protein structure and biological activities of ADAMs.

**Table 1:** Potential functions of human ADAMs*

ADAM	Function/potential function
**Proteolytically inactive**	

ADAM2	Sperm-egg fusion

ADAM7	Sperm maturation

ADAM11	Integrin ligand, neural adhesion, tumour suppressor

ADAM18	Oocyte recognition

ADAM22	Adhesion

ADAM23	Tumour suppressor

ADAM29	Unknown

Proteolytically active*	

ADAM8	Shedding of adhesion molecules, leukocyte receptors, neutrophil infiltration, osteoclast stimulation

ADAM9	α-secretase activity, cellular adhesion

ADAM10	α-secretase activity, shedding of TNF α, EGF, betacellulin, HER2, Notch, and collagen IV, cellular adhesion

ADAM12	Cellular adhesion, shedding of HB-EGF

ADAM15	Cellular adhesion

ADAM17	Release of several growth factor ligands, e.g., TNF-alpha and specific EGFR/HER ligands, cellular adhesion

ADAM19	Unknown

ADAM28	Shedding of IGFBP3

ADAM33	Involved in pathogenesis of gastric cancer via IL-18 secretion

*These functions have been reviewed in detail in refs [1-5].

LPL; lipoprotein lipase, CLL; chronic lymphocytic leukemia, TNFα; tumour necrosis factor-alpha, EGF; epidermal growth factor, HB-EGF; heparin -binding-EGF, IGFBP3; insulin-like growth factor-binding protein 3, IL-18; interleukin-18

### Structure of ADAM Proteins

The generalised structure of an ADAM protein contains 8 distinct domains or regions. In the typical ADAM protein, these domains are a signal domain, a prodomain, a metalloproteinase domain, a disintegrin or integrin-binding domain, a cysteine rich region, an EGF (epidermal growth factor)-like domain, a transmembrane sequence and an intracellular *C*-terminal end [1]. Like most proteases, the ADAMs are initially synthesised as enzymatically-inactive precursor proteins. As with MMPs, this inactive state in most of the ADAMs is due to the interaction of a cysteine residue in the prodomain with the zinc ion at the catalytic site. For protease activation, this prodomain is removed by a furin-like convertase or by autocatalysis, depending on the specific ADAM [1,2]. This cysteine switch mechanism however, does not appear to play a role in maintaining the zymogen state of ADAM17 [6].

Next to the prodomain is the MMP-like domain. Although all ADAMs possess this sequence, only about 50% exhibit protease activity. Thus, of the 21 human ADAMs identified, only 13 are proteolytically active. ADAMs shown to exhibit protease activity include ADAM9, 10, 12, 15, 17, 19, 28 and 33. Currently, protease activity is the best-defined function of ADAMs, with most of the putative substrates currently identified being transmembrane proteins.

Downstream of the MMP domain is the disintegrin domain. This sequence, which is found in all ADAMs binds to integrins, a group of adhesion proteins involved in cell adhesion, migration and cell signalling [7]. It should be stated that most of the work relating to the binding of disintegrins to integrins has been carried out *in vitro *using recombinant disintegrin domains [8]. The biological relevance of these findings are thus unclear.

The function(s) of the remaining domains, i.e., the cysteine-rich region, EGF-like sequence and cytoplasmic remain to be determined. In some of the ADAMs however, the cysteine region has been implicated in regulating protease activity and controlling substrate specificity [9]. The C-terminal domain of ADAM17 has been shown to undergo phosphorylation at different sites including Thr^735 ^and Ser^819 ^[10-14]. Thus, phosphorylation at Thr^735 ^was found to be necessary for ADAM17-catalysed shedding of TGF-alpha [12]. With ADAM15, phosphorylation of the cytoplasmic domain resulted in interaction with several potential signalling proteins, including the Src kinases, Hck and Lek [14]. It was unclear whether or not this interaction led to altered intracellular cell signalling.

### Role of ADAMs in Cancer Formation and Progression

#### Evidence that ADAMs play a causal role in cancer

Studies from cell lines grown in culture, animal models and human malignancies suggest that a number of ADAMs are involved in cancer formation and/or progression [4,5]. Specific ADAMs implicated in these processes include ADAM9, ADAM10, ADAM12, ADAM15 and ADAM17. Of these different ADAMs, the strongest evidence for a role in malignancy exists for ADAM17, which is also known as TACE (tumour necrosis factor alpha converting enzyme). Briefly, the evidence implicating ADAMs in malignancy is as follows [4,5]:

● Inhibition of ADAM17 activity or downregulation of its expression decreased growth of breast cancer cells *in vitro *and reversed their morphological appearance to that approximating normal cells [15].

● Several studies have shown that increased expression of certain ADAMs enhanced *in vitro *invasion, proliferation and promoted tumour formation *in vivo *[16-21], while decreased expression reduced these processes.

● Deficiency of specific ADAMs such as ADAM9, 15 and 17 resulted in decreased growth of heterotopically injected tumour cells in mice models [22,23].

● Correlations exist between between levels of specific ADAMs and parameters of tumour progresion (eg., tumour size, grade, metastasis to local lymph nodes and patient outcome) in human cancers [26-31] and

● Selective inhibitors against certain ADAMs reduce or block tumour cell growth in model system [32-34].

### Mechanisms by which ADAMs play a role in cancer

#### Activation of positively-stimulating pathways

**Figure 1:**
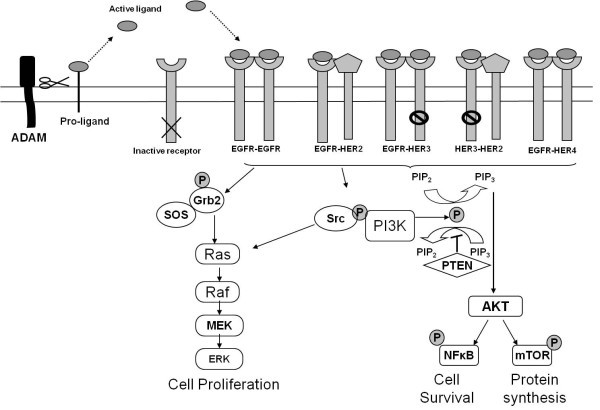
**Mode of action of ADAMs in the activation of EGFR/HER receptor signalling**. ADAMs (primarily ADAM10 and ADAM17) are involved in proteolytic ectodomain shedding of membrane bound ligands. The released ligands (for example, EGF, HB-EGF, TGFα, heregulins) are free to bind to and activate EGFR, HER3 and HER4. Following receptor dimerisation (though HER3 has weak tyrosine kinase activity, its preferred dimerization partner is HER2), downstream signalling through many pathways is activated, including MAPK, PI3K and JAK/STAT.

ADAMs could potentially promote cancer formation and progression using several different mechanisms. One of the most likely of these involves the release or activation of positively-stimulating growth factors (Figure 1). Many of these growth factors are initially synthesised as inactive transmembrane precursor proteins that require conversion to an active state in order to exert maximun activity. Amongst the best-studied growth-stimulating factors that are activated by ADAMs are the EGFR/HER family of ligands. Conversion of these ligands to their active state is primarily mediated by either ADAM10 or ADAM17. Thus, ADAM17 appears to be the physiological sheddase for TGF-alpha, amphiregulin, HB-EGF, and epiregulin. ADAM10, on the other hand, appears to be the major sheddase for the release of EGF and betacellulin [35,36]. In certain situations however, other ADAMs including ADAM8, 9, 12, 17 and 19 can activate one or more of these ligands [37].

The shed form of these ligands binds to one or more of the EGFR/HER family of receptors. Four members of this family exist, i.e., HER1 (c-erbB1), HER2 (c-erbB2), HER3 (c-erbB3) and HER4 (c-erbB4). These 4 receptors have a similar general structure that includes an extracellular domain, a transmembrane domain and an intracellular domain [38-40]. All of these receptors, apart from HER2, can be directly activated following ligand binding. Following homo- or heterodimerisation, downstream signalling from these receptors activates several different pathways including the mitogen-activated protein kinase (MAPK) pathway, the phosphatidylinositol 3-kinase (PI3K) pathway and janus kinase/signal transducer and activator of transcriptional (JAK/STAT) pathway. This signalling results in some of the classical hall markers of malignancy such as enhanced cell proliferation, increased cell motility and increased cell survival [38-40].

Substantial evidence implicating ADAM-mediated growth factor ligand release and EGFR signalling in cancer cell proliferation or migration is now available. Singh et al [41] showed that UV irradiation of skin cancer cells activated ADAM9 and 17 which was followed by amphiregulin shedding, EGFR transactivation and increased cell proliferation. In another study, Zheng et al [21] reported that ADAM17, via ligand release and activation of the EGFR-PI3K-AKT pathway, enhanced *in vitro *breast cancer cell proliferation and invasion. In a further study, Mendelson et al [42] showed that treating mouse embryonic fibroblasts with platelet derived growth factor receptor beta (PDGFRβ) led to activation of ADAM17, release of EGFR ligands and EGFR/ERK signalling. This cascade of events ultimately resulted in enhanced migration.

While shedding of the extracellular domain of the HER ligands results in receptor binding, at least for heparin binding-epidermal growth factor [HB-EGF], it can also lead to translocation of its C-terminal fragment from the cell membrane into the nucleus and regulation of cell proliferation. This translocation of the C-terminal domain of HB-EGF has been shown both in keratinocytes [43] and gastric cancer cells [44] and is thus another possible mechanism by which ADAM-catalysed shedding of growth factors can alter cell proliferation.

#### Inactivation of growth-inhibitory pathways

Inactivation of growth inhibitory signalling systems would be expected to produce the same end result as activation of positively-activating growth factors. One of the best examples of a negatively-acting growth factor is TGFβ which signals via TGFβR1 and TGFβR2 [45]. In normal and early malignant cells, TGFβ inhibits proliferation. In contrast in progressive malignancy, TGFβ promotes proliferation [45]. Recently, ADAM17 was reported to mediate shedding of the type 1 TGFβ receptor [46]. As a result, TGFβ signalling was decreased which in turn led to decreased growth inhibition. According to Liu et al [46], this ADAM17 mediated reduction in growth inhibition complements the growth stimulation, resulting from increased release of the EGFR/HER ligands, see above.

#### Shedding of adhesion proteins

ADAM-mediated shedding of adhesion proteins may also result in increased cell proliferation. Maretzky et al [47] showed that ADAM10 caused shedding of the extracellular domain of cadherin E, which resulted in the translocation of beta-catenin to the nucleus and enhanced proliferation. In other work, Najy et al [48] found that an ADAM15-mediated shed form of cadherin E bound to and activated HER2 in breast cancer cells [48]. The shed form of cadherin E formed a complex with HER2 and HER3, that gave rise to enhanced ERK signalling, which in turn, led to increased proliferation and migration.

It was mentioned above that shedding of cadherin E resulted in increased cell proliferation. As well as enhancing cell proliferation, this shedding might also be expected to weaken cell:cell interaction and thus allow dissociation of potential invasive and metastatic cells in the primary cancer. Such dissociation could potentially place a malignant cell or group of cells on their pathway to metastais. Shedding of other adhesion proteins such as L-selectin, ICAM-1 or VCAM, on the other hand, might be expected to modulate binding of tumour cells to the vasculature wall and thus play a role in the intravasation [i.e., exiting of tumour cells from the vasculature into a distant organ].

#### Potential involvement in mediating angiogenesis

Finally, ADAMs may promote cancer growth and metastasis by mediating angiogenesis or pathological neovascularisation. Angiogenesis is defined as the protrusion and outgrowth of capillary buds and sprouts from pre-existing blood vessels [49]. This process is essential for tumours to grow beyond approximately 2 mm in diameter. Early evidence implicating ADAMs in angiogenesis was the finding of pulmonary hypovascularisation in mice expressing catalytically inactive ADAM17 [50]. More recently, Gooz et al [51] showed that knockdown of ADAM17 expression using siRNA decreased endothelial cell proliferation and invasion *in vitro*. Furthermore, in a mouse model, Weskamp et al [25] reported that deletion of ADAM17 resulted in pathological neovascularisation and reduced growth of injected tumour cells. In this animal model, neither developmental nor vascular homeostasis was affected by the loss of ADAM17. Other ADAMs implicated in pathological neovascularisation include ADAM9 [22] and ADAM15 [23,24].

### ADAMs as Biomarkers in Cancer

Biomarkers are potentially useful in cancer detection (screening and aiding diganosis), asssessing prognosis, upfront predicting likely response or resistance to therapy and monitoring ongoing therapy [for review, see ref. 52]. In recent years, several preliminary reports suggested that a number of different ADAMs may act as cancer biomarkers. This evidence is briefly reviewed below.

#### ADAMs as diagnostic aids in cancer

For aiding cancer diagnosis, a biomarker should be specifically altered in the majority of patients with a specific malignancy or premalignant condition. Furthermore, it should be measurable in a readily available fluid such as serum or urine. In recent years, a number of ADAMs have been detected in these fluids from patients with cancer. One of the first ADAMs shown to have diagnostic potential was ADAM12 in breast cancer. Using Western blotting, Roy et al [53], reported that urinary levels of ADAM12 were significantly increased in patients with breast cancer vis-à-vis a healthy control group. Furthermore, the proportion of patients with high levels of this ADAM was significantly greater in the breast cancer patients than in the healthy controls. Levels were disease stage-related, progressively increasing from patients with in situ disease, to those with locally invasive disease to those with metastatic disease. Using logistic regression analysis, the authors calculated that the predictive probability of the presence of breast cancer was ≥ 80%, when levels of ADAM12 exceeded 40 arbitrary units [53].

In a follow-up study to above, Pories et al [54] found that urinary ADAM12 levels were also increased in women with putative premalignant lesions of invasive breast cancer such as atypical hyperplasia and lobular carcinoma in situ, compared to levels in healthy controls. This finding, if confirmed, suggests that measurement of ADAM12 in urine could identify women at increase risk of developing breast cancer. Clearly, these preliminary but promising findings, require confirmation in larger studies. It should be stated that none of the available serum markers for breast cancer are increased in patients with early disease and are thus of little value in identifying women at increased risk of developing this malignancy [55].

As well as breast cancer, ADAM12 has also been found to be elevated in urine from patients with bladder cancer, compared with healthy control subjects [56]. Indeed, measurement of ADAM12 appeared to be a more sensitive diganostic marker for bladder cancer that standard cytology. Although levels were increased in patients with early stage disease, including those with superficial non-invasive disease and superficial invasive disease, concentrations tended to be higher in those with the largest invasive tumours. In the small number of cases studied, urinary ADAM12 levels decreased following surgical removal of the bladder cancer but increased again with recurrent disease [56]. This latter finding suggests that measurement of urinary ADAM12 may be suitable for monitoring patients with bladder cancer.

One of the first ADAMs shown to be elevated in serum from patients with cancer was ADAM28 [57]. Using ELISA, Kuroda et al [57] found that serum levels of this ADAM in patients with non-small cell lung cancer were approximately 5-fold greater than levels in a healthy control group. As with urinary levels of ADAM12 in breast cancer, serum levels of ADAM28 increased progressively with increasing disease stage. Interestingly, the diagnostic acuracy of ADAM28 appeared to be higher than that of one of the establised marker for non-small cell lung cancer, i.e., carcinoembryonic antigen (CEA).

#### ADAMs as prognostic markers

Prognostic markers are important in the management of patients with cancer as they help avoid the overtreatment of indolent disease and undertreatment of aggressive cases. Ideally, a new biological prognostic marker should provide additional or independent information to that available from the conventional factors such as tumour size, tumour grade and metastasis to local lymph nodes. New prognostic markers are most urgently needed for cancers such as breast and prostate cancer. In breast cancer, prognostic markers may help identify those patients whose prognosis is so good that they are unlikely to benefit from receiving adjuvant chemotherapy. The corollory is that the same marker(s) can help identify patients with aggressive disease that may derive benefit from receiving such therapy. As certain ADAMs have been implicated in tumour development and progression, it is not surprising that they have been investigated for potential prognostic impact in patients with cancer.

One of the best validated ADAMs for predicting patient outcome is ADAM17 in breast cancer. Using ELISA, McGowan et al showed that patients with breast cancers expressing high levels of ADAM17 protein had significantly shorter overall survival compared to those with low expression of the protein [31]. Importantly, the prognostic impact of ADAM17 was independent of tumour size, grade and lymph node status. Although the ELISA used in this study detected both the precursor and active forms of ADAM17, a previous study found that the active form was more associated with breast cancer progression than the precursor form [16]. As well as ADAM17 protein, high expression of ADAM17 mRNA was also found to predict adverse outcome in patients with breast cancer [15].

Another ADAM associated with outcome in patients with breast cancer is ADAM15. Four isoforms or variants of this ADAM have been described, ADAM15-A, ADAM15-B, ADAM15-C and ADAM15-D. These different forms of ADAM15 have been shown to have different effects *in vitro *[29]. Thus, ADAM15-A was found to increase cell invasion, migration and adhesion, while ADAM15-B was shown to decrease adhesion. Although these 2 variants had different effects on adhesion *in vitro*, high expression of both predicted shortened relapse-free survival in lymph node-negative breast cancer patients. ADAM15-C, on the other hand, correlated with improved relapse-free survival in lymph node-positive but not in lymph node-negative patients.

One of the cancers for which new prognostic markers are most urgently required is prostate cancer. Although prostate cancer is the most common malignancy affecting males, most of these tumours are indolent and never progress to a symptomatic stage. However, a minority are aggressive and rapidly progress causing morbidity and mortality. A major everyday problem in the management of men with newly diagnosed prostate cancer is therefore differentiating men with indolent disease from those with life-threatening disease.

Using immunohistochemistry, Fritzche et al [28], showed that increased expression of ADAM9 in prostate cancer was significantly associated with shortened relapse-free survival as measured by increasing serum PSA levels. As with ADAM17 in breast cancer, the prognostic impact of ADAM9 in prostate cancer was independent of the conventionally used factors for this malignancy such as tumour size, Gleason grade and preoperative PSA level. This independent prognostic impact of ADAM9 was found in both the total population of patients investigated as well as in those treated with anti-androgens [28]. Other malignancies for which ADAM9 has also been shown to have prognostic value include renal [27] and pancreatic cancers [58].

#### ADAMs as therapy predictive markers

Predictive markers are factors that are associated with upfront response or resistance to a particular therapy [59]. Predictive markers are important in the management of cancer patients as tumours of the same histological type or tissue of origin vary widely in their response to most available systemic therapies. Ideally, therefore, markers should be available that predicts likely response. Markers that predict resistance however, may also be helpful. In this latter situation, if available, patients could receive an alternative therapy that may be more beneficial. If an effective alternative therapy is unavailable, these patients could volunteer to participate in clinical trials evaluating new therapies or they could make an informed decision to avoid the needless costs and toxicity of likely ineffective therapy [59].

In one of the few studies carried out to date on ADAMs as therapy predictive biomarkers, Siewerts et al [60] reported that high levels of mRNA for ADAM9 and 11 but not for ADAM10 or ADAM12 were associated with increased benefit from tamoxifen in patients with recurrent breast cancer [60]. This finding was especially true for patients whose primary tumour contained large amounts of stroma. ADAM9 but not ADAM11 provided independent predictive information over estrogen receptors, progesterone receptors, menopausal status and dominant site of relapse.

### Summary and Conclusion on ADAMs as Cancer Biomarkers

Although the above studies indicating a cancer biomarker potential for different ADAMs are promising, they all require confirmation in larger and prospective studies. For evaluating a potential cancer diagnostic role for the ADAMs, it is important that control samples are taken from subjects with a relevant benign disease rather from a healthy population. For studies evaluating a prognostic impact, homogenously-managed groups of patients should be investigated. Such a study is best carried out prospectively although a sufficiently high-powered retrospective analysis lacking bias should also provide reliable results. Predictive biomarkers are most conveniently evaluated in either the neoadjuvant or advanced disease settings, i.e., where measurable disease is present. In the adjuvant setting, predictive markers should be investigated as part of a randomised trial in which the marker is used to determine response in the treatment arm while a potential prognostic value can be evaluated in the control arm without systemic treatment.

### ADAMs as Therapeutic Targets for the Treatment of Cancer

Since considerable evidence from model systems suggest that specific ADAMs are causally involved in cancer formation and progression, it might be expected that inhibition of these proteases could be used to treat cancer. At least 4 potential approaches exist to block ADAM protease activity. These include use of low molecular weight synthetic inhibitors [see below], purified or synthetic forms of ADAM prodomains [61,62], modified TIMPs [63,64] and monoclonal antibodies [65]. Of these potential approaches, only the use of low molecular weight synthetic inhibitors has been subjected to detailed investigation.

Most of the low molecular weight ADAM inhibitors use hydroxamate as the zinc binding group and were designed to bind to the MMP-like catalytic site [66-69]. Although the majority of those described, inhibited a number of MMPs as well as some ADAMs, a small number were relatively selective for specific ADAMs, especially ADAM10 and/or ADAM17 [32-34,70-75] (Table 2). Of the compounds listed in Table 2, the most widely investigated for anticancer activity are INCB3619 and INCB7839 [Incyte Corporation, Wilmington, DE] [32-34,70-72].

**Table 2:** Selective ADAM inhibitors

Inhibitor	Target ADAM	Company	Refs
INCB3619	10, 17	Incyte, Wilmington, DE	[32-34]

INCB7839	10,17	Incyte	[34,70-72]

WAY-022	17	Wyeth-Aherst, Pearl River, NY	[73]

GI254023X	10	Glaxo Smith Kline	[74]

GW280264X	10,17	Glaxo Smith Kline	[74]

TMI-2	17	Pfizer	[75]

KB-R7785	12	Organon, Osaka, Japan	[80]

INCB3619 is an orally-active low molecular weight molecule that selectively inhibits ADAM10 and ADAM17 with low IC_50 _values [14 and 22 nmoles/L, respectively] [33]. It has been shown to inhibit tumour cell growth in several different preclinical models. Thus, in an early study, with non small cell lung cancer [NSCLC] cells in culture, INCB3619 was found to block release of the HER3 ligand, heregulin, rendering these cells sensitive to the EGFR inhibitor, gefitinib [32]. Also, using NSCLC cell, INCB3619 increased apoptosis and reduced the apoptotic threshold for response to paclitaxel [32]. Consistent with this finding, the inhibitor decreased tumour growth and enhanced the therapeutic benefit of paclitaxel in a xenograft model of these cells.

As well as lung cancer, INCB3619 has also been shown to block the growth of breast cancer. Thus, INCN3619 was shown to synergise with paclitaxel in inhibiting growth of breast cancer in a xenograft model [32]. In a different study, although the addition of this compound to MCF-7 breast cancer cells *in vitro *resulted in minimal growth inhibition, when combined with the dual EGFR/HER2 tyrosine kinase inhibitor, GW2974 (Sigma Aldrich), synergistic growth inhibition was observed [33]. The combination of INCB3619 and GW2974 also gave rise to decreased phosphorylation of ERK and AKT, suggesting blockage of the MAPK pathway. Using a xenograft breast cancer model, an inhibitor related to INCB3619, i.e., INCB7839 was found to decrease tumour volume [34]. However, when combined with the tyrosine kinase inhibitor, lapatinib, complete inhibition of tumour growth was observed. An important finding with the animal models investigated was that administration of INCB3619, in contrast to previous studies with MMP inhibitors [76,77], did not appear to induce musculoskeletal side effects [33].

INCB7839 is currently undergoing early clinical trials in HER2-positive advanced breast cancer patients [71,72]. Preliminary results suggest that this drug is generally well tolerated with no significant adverse effects that might be expected from inhibition of MMPs (musculoskeletal side effects) or EGFR-related kinases (skin rash). Furthermore, there was no evidence of drug-induced increases in liver enzymes, bone marrow toxicity or increase in cardiomyopathy. In a recently reported abstract [72], administration of INCB7839 and trastuzumab to 51 patients with advanced HER2-positive breast cancer induced response in 13/26 (50%) evaluable patients. In 14 patients where the INCB7839 plasma concentration exceeded the IC_50 _for HER2 cleavage, response was obtained in 9 (64%). Previous studies had shown that administration of trastuzumab monotherapy to patients with advanced breast cancer induced response rates of approximately 14-35% [78,79]. Thus, it would appear that the addition of INCB7839 to trastuzumab increased efficacy. Phase III clinical trials however, will be necessary to confirm this finding.

#### Potential side effects from anti-ADAM treatments

Most of our information on the potential side-effects of anti-ADAM inhibitors has been derived from clinical trials involving the use of ADAM17 inhibitors to treat patients with rheumatoid arthritis [68,69]. Unfortunately, most of these studies were limited to phase I and phase II trials because of lack of efficacy and/or hepatotoxicity. The origin of the hepatotoxicity is unclear. However, as mentioned above, early results from phase I/II trials with the dual ADAM10/17 inhibitor INCB7839 do not suggest major toxicity problems with this agent. As mentioned above, a particularly encouraging finding with INCB3619 and INCB7839 is that they do not appear to cause musculoskeletal side effects [33], which was a major problem with the early metalloproteinase inhibitors investigated [76,77]. Continued caution however, with respect to toxicity, will be necessary, especially as ADAM10 and ADAM17 act on wide variety of membrane proteins [1,2].

## Competing interests

The authors declare that they have no competing interests.

## Authors' contributions

MJD researched the literature, conceived and wrote the manuscript. PMcG designed and formatted Figure 1. All authors read, edited and agreed with the content.
